# Single‐Molecule Protein Profiling Using Nanopores and Dimeric Aptamer‐Modified DNA Carriers

**DOI:** 10.1002/anie.202505902

**Published:** 2025-08-15

**Authors:** Xiaoyi Wang, Yaxian Liu, Ren Ren, Joshua B. Edel, Aleksandar P. Ivanov

**Affiliations:** ^1^ Department of Chemistry Imperial College London Molecular Sciences Research Hub White City Campus 82 Wood Lane London W12 0BZ UK; ^2^ Department of Metabolism Digestion and Reproduction Imperial College London Hammersmith Campus Du Cane Road London W12 0NN UK; ^3^ Picower Institute for Learning and Memory Massachusetts Institute of Technology (MIT) Cambridge MA 02139 USA

**Keywords:** Aptamers, Diagnostics, Molecular carriers, Nanopores, Single‐molecule protein detection

## Abstract

Single‐molecule protein profiling offers unparalleled sensitivity for detecting rare biomarkers, with significant potential for early disease diagnosis and monitoring. Nanopore sensors enable the detection of proteins in solution and are well‐suited for clinical applications due to their high sensitivity and throughput. Here, we introduce a nanopore‐based detection strategy utilising an aptamer‐protein‐aptamer sandwich structure to enhance protein detection further. The aptamer probe is extended with a double‐stranded DNA carrier to enhance protein transport and detection efficiency. Upon protein binding, dimerization of the DNA carriers occurs, allowing charge‐based classification of bound versus unbound states. Additionally, the sandwich complex produces a characteristic subpeak within individual nanopore events, with subpeak height correlating to protein size. This approach enables selective detection of dimeric proteins, including vascular endothelial growth factor (VEGF) and platelet‐derived growth factor (PDGF), with sub‐picomolar detection limits. In principle, it can be adapted for any protein with two distinct aptamer‐binding sites, as demonstrated with thrombin. This method also facilitates real‐time monitoring of ligand‐induced receptor dimerization in complex biological fluids, offering a powerful tool for potential single‐molecule‐based diagnostics.

## Graphical abstract

We developed a nanopore‐based biosensor for single‐molecule protein profiling using aptamer‐modified DNA carrier dimerization strategy, enabling selective and ultrasensitive detection of proteins and protein complexes. This approach promises advances in early‐stage clinical diagnostics and precision medicine.

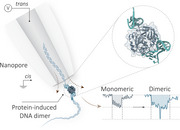

## Introduction

Early detection of diseases, particularly in cases such as cancer and neurodegenerative disorders, holds significant potential to enhance treatment efficiency, life quality, and survival rates.^[^ ^1^ ^]^ However, biomarkers in early disease stages often exhibit low abundance and dynamic, transient nature compared to advanced stages, making them elusive for detection.^[^ ^2^ ^]^ Conventional detection methods, such as lateral flow assays (LFAs) and enzyme‐linked immunosorbent assays (ELISAs), often lack the sensitivity and specificity required for multiplexing diverse protein biomarkers.^[^ ^3^, ^4^ ^]^ In contrast, single‐molecule detection promises to identify single target biomarkers at very low concentrations and sample volumes, providing new opportunities for clinical diagnostics.^[^ ^5^, ^6^, ^7^ ^]^ Among these approaches, nanopore technology provides a powerful platform for rapidly identifying and quantifying disease‐related biomarkers at the single‐molecule level.^[^ ^8^ ^]^ By applying an electric field, this method monitors ionic current changes as molecules translocate through the nanopore, allowing discrimination of biomolecules, including nucleic acids, proteins, and complexes, based on their size, while event frequency provides an estimate of their concentration. However, nanopore detection in complex biofluids is often hindered by limited selectivity and multiplexing capacity due to the presence of multiple background biomolecules.^[^ ^9^ ^]^ Furthermore, achieving reliable high signal‐to‐noise ratios and detection robustness in physiological environments remains challenging, as nonspecific interactions, molecular crowding, and ionic strength fluctuations can substantially interfere with signal interpretation.^[^ ^10^, ^11^ ^]^

To address these selectivity challenges, molecular carrier‐based strategies have been developed for nanopore protein sensing.^[^ ^9^ ^]^ These molecular carriers typically feature a suitably charged backbone, which could be composed of DNA,^[^ ^12^, ^13^ ^]^ charged peptides,^[^ ^14^ ^]^ or metallic nanoparticles.^[^ ^14^ ^]^ Driven by strong electrokinetic forces, the charged carriers facilitate the transport of proteins through the nanopores, thus enhancing the overall detection efficiency. Among the range of carriers available, DNA carriers have attracted substantial attention due to their advantages, such as rigid double‐helix structures, programmable sequences, and flexible origami scaffolds. Aptamers, short single‐stranded nucleic acids that bind specific targets with high affinity, have emerged as powerful recognition elements in nanopore sensing platforms,^[^ ^15^, ^16^, ^17^ ^]^ particularly when integrated with DNA molecular carriers.^[^ ^13^, ^18^, ^19^, ^20^, ^21^ ^]^ For example, a recent study demonstrated the simultaneous and multiplexed detection of over 40 biomarkers using DNA‐barcoded molecular carriers.^[^ ^22^ ^]^ The success of this method hinges on the ability of DNA carriers to selectively capture and transport biomarkers via modified recognition elements. In protein detection, the target protein is typically tethered to the DNA carrier through covalent or non‐covalent interactions. The presence of the protein induces a distinct subpeak within the DNA translocation signal, serving as a clear indicator of successful binding.^[^ ^13^ ^]^

**Figure 1:**
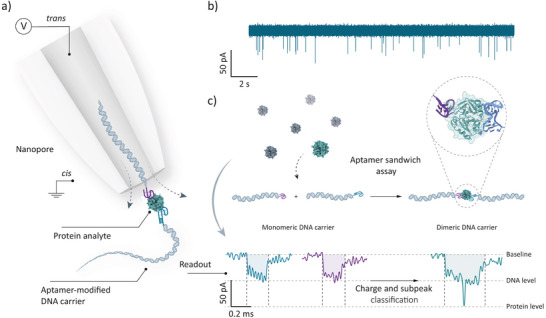
Nanopore‐based protein detection using aptamer‐protein‐aptamer sandwich structures. a) Schematic of the experimental setup for single‐molecule detection using nanopipettes. The protein binds to two aptamer‐modified DNA carriers, generating a dimeric complex that indicates the presence of the target protein. The protein and DNA carriers are added into the internal bath of the nanopipette (*trans* side) with the measuring buffer (100 mM KCl, 10 mM Tris‐EDTA pH 8.0), where a working Ag/AgCl electrode is situated. The external bath (*cis* side), where the reference Ag/AgCl electrode is placed, is filled with the measuring buffer without analytes. b) A typical current trace shows current‐conductive events of DNA translocation under the application of a bias of −300 mV. c) An example of a thrombin protein with two different aptamer‐binding sites (PDB: 5EW2) can form a sandwich structure with designed DNA carriers. Upon the successful formation of such structures, translocation events exhibit distinctive characteristics, including double dwell time and equivalent charge, as well as a discernible subpeak at the midpoint of the event.

Here, we present a nanopore‐based protein profiling strategy that utilizes an aptamer‐protein‐aptamer sandwich structure on a DNA carrier, as illustrated in Figure 1. In the presence of target proteins, a single protein molecule binds two aptamer‐modified DNA carriers, forming a sandwich complex and converting carrier monomers into dimers. This detection method operates efficiently under low‐salt conditions (100 mM KCl), comparable to physiological environments, enabling single‐protein detection sensitivity while preserving protein binding. Protein analytes can be identified using a straightforward classification approach based on a single parameter: equivalent charge (peak area of individual events), eliminating the need for subpeak analysis. This strategy broadly applies to proteins with dual aptamer‐binding sites, as demonstrated by the detection of thrombin using HD1‐ and HD22‐modified carriers. Notably, the HD1‐thrombin‐HD22 sandwich complex induces a subpeak within heterodimeric DNA events, resembling observations at higher salt concentrations.

Additionally, this approach is well‐suited for detecting protein dimers. Using vascular endothelial growth factor (VEGF) and platelet‐derived growth factor (PDGF) as model proteins, we demonstrate that two identical aptamer‐modified carriers facilitate sandwich complex formation, enabling the detection of dimeric proteins at low analyte concentrations. Furthermore, this strategy extends to monitoring hepatocyte growth factor (HGF)‐induced c‐Met receptor dimerization and its inhibition by anti‐HGF antibodies. Importantly, subpeak height correlates consistently with protein size, highlighting the potential of this method for precise protein biomarker identification. Measurements performed in human serum confirm the compatibility of this method with real clinical samples, further highlighting its clinical applicability.

## Results and Discussion

**Figure 2:**
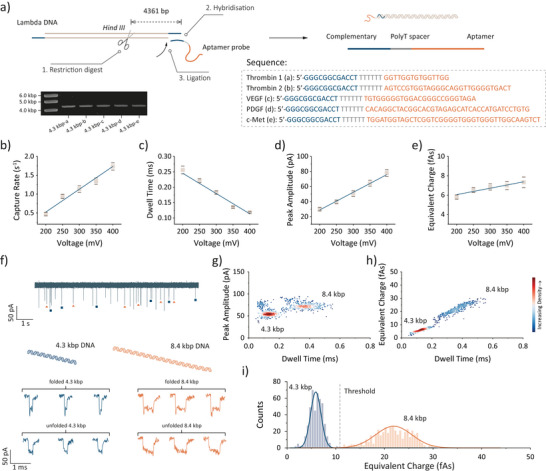
DNA Carrier preparation and charge classification. a) 4.3 kbp DNA carriers were cleaved from λ‐DNA using, followed by hybridization and ligation of the aptamer probe to the carrier. The aptamer probe includes a sequence complementary to the 12‐base sticky overhang of the 4.3 kbp carrier, a polyT spacer, and a protein aptamer sequence. The final products were characterized by gel electrophoresis. Plots of b) capture rate, c) dwell time, d) peak amplitude, and e) equivalent charge are shown along with the fitting curve. The error bars represent one standard deviation of at least three independent experimental repeats. f) A representative current trace of nanopore translocation for a mixture of 100 pM 4.3 kbp and 100 pM 8.4 kbp DNA. 4.3 kbp and 8.4 kbp DNA were directly purified from λ‐DNA HindIII digest and BstEII digest, respectively. Typical events are shown corresponding to 4.3 and 8.4 kbp DNA at folded and unfolded conformations, respectively. Density scatter plots of g) peak amplitude against dwell time and h) charge against dwell time for the mixture of 4.3 and 8.4 kbp DNA (*n* = 892). i) Distribution of the charge showing that 4.3 and 8.4 kbp (*n* = 892) DNA are easy to differentiate by setting a threshold of charge. Nanopore experiments were performed in the measuring buffer containing 100 mM KCl and 10 mM Tris‐EDTA pH 8.0 under a bias of −300 mV.

Nanopore experiments were conducted using single‐barrel quartz nanopipettes that were fabricated through a laser‐assisted pulling process.^[^ ^14^ ^]^ The nanopore size was characterized using scanning electron microscopy (SEM) and conductance measurements, Figure S1. Electrical measurements were conducted in a buffer solution containing 100 mM KCl and 10 mM Tris‐EDTA (pH 8.0), unless otherwise specified. The current‐voltage (*I‐V*) curves exhibited characteristic ionic current rectification, consistent with the use of the nanopipette, under these conditions.^[^ ^23^ ^]^ DNA carriers and protein analytes were introduced into the nanopipette's interior, where a working Ag/AgCl electrode was positioned. A reference Ag/AgCl electrode was placed in the external bath, containing the same buffer. Ionic current traces were recorded using a high‐bandwidth amplifier (Chimera Instruments, VC100) at a 1 MHz sampling rate, followed by low‐pass filtering at 30 kHz. Signal quality was assessed using power spectral density (PSD) analysis, confirming low noise levels, Figure S2. DNA translocation from the inside of the pipette to the bath exhibited a current increase when subjected to a negative bias. No translocation events were detected in the opposite direction. Phage lambda DNA (λ‐DNA) was selected as the carrier due to its well‐characterized sequence and 12‐base sticky overhangs at both the 5′ and 3′ termini, allowing functional oligonucleotide modification via hybridization. As illustrated in Figure 2a, λ‐DNA was digested using the restriction endonuclease HindIII, yielding a 4.3 kbp DNA fragment with a 12‐base overhang at one end. This fragment was hybridized and ligated with an aptamer probe, followed by gel extraction for purification. Each aptamer probe comprised three key components: i) a sequence complementary to the sticky end of the DNA carrier, ii) a polyT spacer sequence, and iii) a specific region for protein binding. For proteins with two distinct aptamer‐binding sites (e.g., thrombin), two carriers with different aptamer probes were prepared separately, whereas for dimeric proteins, only one carrier was required. Detailed sequence information is provided in Table S1. The aptamer‐modified 4.3 kbp DNA carriers were characterized via gel electrophoresis and UV–Vis spectroscopy (Figure S4).

Control experiments with DNA carriers were performed under varying applied voltages, ranging from −200 to −400 mV, as shown in Figure S5. The capture rate, derived from the distribution of interval times (∆t) between consecutive translocation events, exhibited a linear voltage dependence (Figure 2b), indicating a diffusion‐limited regime governing the capture of DNA carrier molecules.^[^ ^24^ ^]^ The dwell time as a function of voltage followed a linear decay (Figure 2c), with mean dwell times ranging from 256 ± 10 to 119 ± 2 µs at 200 and 400 mV, respectively, confirming the suitability of a 30 kHz low‐pass filter for processing single molecule signals reliably.^[^ ^25^ ^]^ As expected, the mean peak amplitude increases linearly with voltage, ranging from 30.0 ± 2.3 to 77.8 ± 4.5 pA, Figure 2d. To balance capture rate and signal‐to‐noise ratio (SNR), an applied voltage of −300 mV was selected for further nanopore measurements. The integrated area of individual events, referred to as the “equivalent charge,” showed a gradual increase with voltage (Figure 2e). This equivalent charge is associated with the number of counterions associated with the DNA molecule,^[^ ^26^ ^]^ which could be used to classify DNA size.

Traditionally, successful protein binding is confirmed by the appearance of a distinct subpeak in the signal.^[^ ^12^ ^], [^ ^13^ ^]^ However, this method struggles to detect smaller proteins (<15 kDa) due to their low SNR, making resolution challenging. Additionally, partial folding of DNA carriers can generate similar subpeaks, leading to potential false positives and complicating data interpretation. We employed CUSUM+, a variant of the cumulative sum statistical algorithm for single‐event classification Figure S6.^[^ ^27^ ^]^ For each translocation, we calculated the equivalent charge from the integration area of individual events.^[^ ^28^ ^]^ We used 4.3 kbp and 8.4 kbp DNA fragments as monomeric and dimeric carriers, respectively. As shown in Figure 2f, the 4.3 kbp fragments translocated faster than the 8.4 kbp DNA. However, due to folding, the dwell time and peak current distributions did not exhibit two distinct populations; instead, overlapping distributions were observed (Figure S7a,b). To better resolve these populations, we constructed 2D density scatter plots of peak amplitude versus dwell time (Figure 2g), which revealed four distinct clusters corresponding to the folded and unfolded states of 4.3 and 8.4 kbp DNA. While visually distinguishable, precise classification required the development of a dedicated algorithm.^[^ ^29^ ^]^ In contrast, the scatter plot of equivalent charge versus dwell time (Figure 2h) exhibited two well‐separated populations, allowing differentiation using a single charge threshold. The distribution histogram of equivalent charge (Figure S7) further confirmed these populations, each containing two subpopulations representing folded and unfolded DNA. Notably, folded DNA exhibited lower charge values than unfolded DNA of the same length, likely due to reduced surface area and fewer bound counterions. By applying a straightforward charge‐based classification, we successfully distinguished 4.3 kbp from 8.4 kbp DNA, demonstrating the feasibility of using this method to differentiate DNA monomers and dimers for protein target detection.

### Thrombin Detection by Heterogenous Dimerization of DNA Carriers

Having established the ability to distinguish between monomeric and dimeric DNA, we applied this approach to protein detection using two distinct aptamer‐modified DNA carriers. As a model protein, we selected human alpha thrombin (α‐thrombin), a 36.7 kDa protein with an isoelectric point (pI) of 7.0–7.4.^[^ ^13^ ^]^ α‐Thrombin is a key biomarker in cardiovascular diseases, tumor regulation, and coagulation cascades,^[^ ^30^ ^]^ and also acts as a neurotoxin produced by injured brain endothelial cells, making it critical for tracking Alzheimer's disease progression.^[^ ^31^ ^]^ Early detection of thrombin‐related diseases requires high selectivity and sensitivity to detect trace levels in complex biological fluids such as blood and cerebrospinal fluid (CSF). Aptamer‐based sandwich biosensors have been widely explored for thrombin detection, employing two G‐quadruplex aptamers that bind distinct exosites of thrombin.^[^ ^32^ ^]^ These include the 15‐mer thrombin‐binding aptamer (TBA, HD1) with a dissociation constant (*K*_d_  =  20.2 nM) and the 29‐mer aptamer (HD22) with a stronger affinity (*K*_d_  =  3.5 nM). Notably, dimerization of HD1 and HD22 enhances binding affinity by order of magnitude due to the avidity effect.^[^ ^33^ ^]^

**Figure 3:**
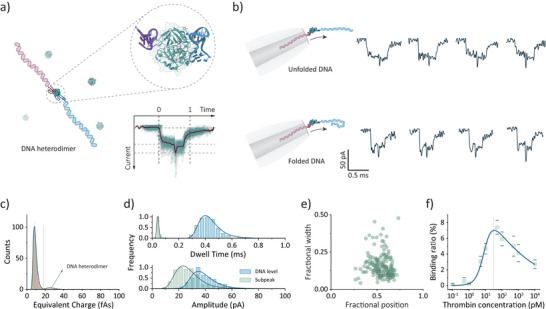
Nanopore detection of thrombin using dimerization of aptamer‐modified DNA carriers. a) Schematic of the thrombin structure sandwiched by HD1‐ and HD22‐ modified DNA carriers (PDB: 5EW2). Inset: overlay of 88 dimeric events formed by the HD1‐thrombin‐HD22 sandwich structure. b) Diagrams of nanopore translocation for unfolded and partially folded dimeric events. Nanopore experiments were performed using 100 pM thrombin with 100 pM of each carrier in 100 mM KCl measuring buffer under a bias of −300 mV. c) Histogram of charge showing two populations that correspond to monomeric DNA carriers and dimeric DNA carriers, respectively, in the presence of 50 pM thrombin (*n* = 4466). d) Distributions of dwell time and peak amplitude for extracted subpeaks and DNA levels (*n* = 88). e) Scattering points of fractional width (defined as the ratio of subpeak width to the width of DNA level) against the fractional position, where the subpeak maxima were located within the event for extracted dimeric events (*n* = 88). f) Binding assays of 100 pM of each carrier in the presence of thrombin ranging from 100 fM to 10 nM. The binding curve was fitted with a biphasic Hill model and the *K*_d_ value was determined to be about 9.1 pM. The error bars represent one standard deviation of at least three independent experimental repeats.

Control experiments with thrombin alone under identical measurement conditions showed no detectable translocation events, Figure S8. Given its small size and near‐neutral charge at physiological pH, thrombin is challenging to detect directly. To facilitate selective detection, we employed 4.3 kbp DNA carriers separately modified with HD1 and HD22 aptamers (Figure 3a). These carriers (100 pM each) were incubated with 100 pM thrombin at 37 °C for 2 h before nanopore measurements. Upon thrombin addition, protein‐induced dimerization events can be detected. These dimeric 8.6 kbp DNA events, distinguishable from the folded and unfolded states of 4.3 kbp DNA monomers, exhibited extended dwell times and increased peak areas (Figure S9). A notable observation in these results is the presence of a subset of dimeric events featuring a sharp subpeak localized within the central region (Figure 3a,b). This subpeak likely originates from the formation of aptamer‐protein‐aptamer complexes. While proteins typically do not generate conductive current enhancements at low salt concentrations,^[^ ^34^ ^]^ their binding to the highly charged G‐quadruplex aptamers could increase local charge density within the nanopore sensing region, leading to such enhancements.^[^ ^35^ ^]^

To quantify thrombin concentration, a charge threshold of 18 fAs was applied to the charge distribution (Figures 3c and S10), effectively distinguishing monomeric events (mean charge: 7.7 ± 0.4 fAs) from dimeric events (mean charge: 23.3 ± 1.7 fAs) within three standard deviations of the mean dimeric distribution. Notably, the charge of dimeric DNA does not precisely double that of monomeric DNA, as it reflects the net difference between ions bound to the DNA molecules and those diffusing in the solution rather than following a simple linear relationship. Subsequent analysis examined the subpeaks within dimeric events (Figure 3d). The dwell time of these subpeaks was significantly shorter (mean: 32 µs) compared to the main DNA levels (mean: 432 µs), aligning with the small proportion of the aptamer‐protein‐aptamer sandwich complex within the entire carrier structure. The subpeak amplitude averaged 23.1 pA, slightly lower than that of unfolded DNA (34.8 pA), suggesting that the charge enhancement caused by aptamer‐protein complex is less than that of an unfolded double‐stranded DNA within the nanopore sensing region. The lengths of individual events were standardized to begin at 0 and end at 1 (Figure 3a). A scatter plot of fractional width (subpeak width relative to the DNA event width) versus fractional position (subpeak maxima along the event waveform) revealed a preference for subpeaks around position 0.55 rather than the exact midpoint (Figure 3e). This shift is attributed to the translocation dynamics of DNA, which initially slows before accelerating near the event's end, consistent with previous studies.^[^ ^36^ ^]^

A binding assay was conducted with thrombin concentrations ranging from 100 fM to 10 nM, following the same protocol. For each concentration, the binding ratio was calculated as the number of dimeric events divided by the total number of events. The resulting data were used to construct a binding curve, which exhibited a two‐step sigmoidal shape, Figure 3f. At low thrombin concentrations (0.1–50 pM), thrombin facilitates aptamer‐protein‐aptamer sandwich formation, leading to DNA dimerization. However, as thrombin concentration increases, it preferentially binds to a single DNA carrier, thereby preventing dimer formation. This phenomenon, known as the hook effect, arises when excess analyte saturates available binding sites, a behavior commonly observed in sandwich immunoassays.^[^ ^37^ ^]^ For detection purposes, the primary focus is on the first phase of the binding curve, which correlates with the total concentration of monomeric DNA carriers. When the carrier concentration was set to 200 pM, *K*_d_ was determined to be 9.1 pM which is lower than values reported from bulk Surface Plasmon Resonance measurement.^[^ ^33^ ^]^ By analyzing the linear region at low concentrations, the limit of detection (LOD), defined as the concentration of three standard deviations (3σ) above background noise (blank measurements), was found to be 0.20 pM (Figure S11).

### Detection of Dimeric Proteins by Using Identical DNA Carriers

A more direct detection strategy involves using two identical DNA carriers to recognize protein dimers. Protein dimerization, a fundamental protein‐protein interaction, plays a key role in regulating physiological and pathological processes, including enzymatic activation, transcriptional regulation, signal transduction, and disease progression.^[^ ^38^ ^]^ To validate this approach, we selected two homodimeric proteins as proof of concept: vascular endothelial growth factor (VEGF; 42 kDa; pI of 7.45)^[^ ^39^ ^]^ and platelet‐derived growth factor (PDGF; 30 kDa; pI of 9.8).^[^ ^40^ ^]^ Both proteins are essential for angiogenesis and vascular development and serve as key biomarkers for various diseases. VEGF is particularly significant in tumor pathophysiology, acting as a potent inducer of angiogenesis that promotes tumor growth and metastasis.^[^ ^41^ ^]^ PDGF, on the other hand, is strongly implicated in cancer progression, vascular disorders, and fibrotic diseases.^[^ ^42^ ^]^

**Figure 4:**
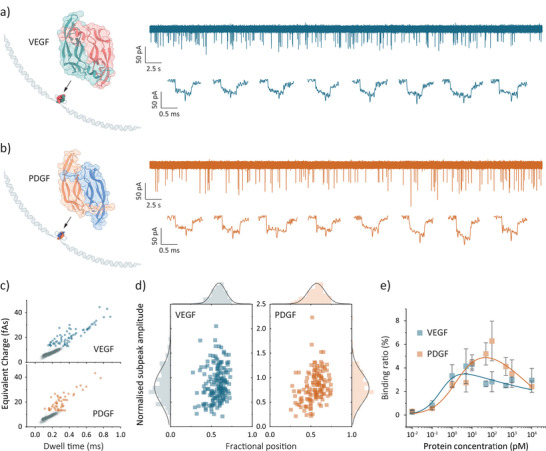
Nanopore detection of dimeric proteins using aptamer‐modified DNA carriers. Schematic of the dimeric protein bound to two identical aptamer‐modified DNA carriers for a) VEGF and b) PDGF. Representative current traces and typical dimeric events were collected with 100 pM protein concentration and 200 pM carrier concentration in the measuring buffer under a bias of −300 mV. c) Scatter plots of charge against dwell time for VEGF (*n* = 3951) and PDGF (*n* = 2299), where the dimeric events over the charge threshold were highlighted in blue and orange, respectively. d) Scattering points of normalized subpeak amplitude (defined as the ratio of subpeak amplitude to the height of DNA level) against the fractional position for extracted dimeric events, along with the statistical histograms, for VEGF (*n* = 168) and PDGF (*n* = 139). e) Binding assays of 200 pM DNA carriers in the presence of VEGF and PDGF ranging from 10 fM to 10 nM. The binding curves were fitted with a biphasic Hill model, and the *K*_d_ value was determined to be about 0.3 and 1.8 pM for VEGF and PDGF, respectively. The error bars represent one standard deviation of at least three independent experimental repeats.

As shown in Figure 4a,b, protein dimers can be detected using two identical aptamer‐modified DNA carriers, which form an aptamer‐protein‐aptamer sandwich structure. Specifically, the VEGF aptamer (V7t1) with an affinity of ≈1.4 nM, was used to capture VEGF‐165 (38.4 kDa),^[^ ^43^ ^]^ while a previously reported 39‐mer PDGF aptamer (affinity ≈0.1 nM) was selected for detecting PDGF‐BB (24.6 kDa), a PDGF family variant.^[^ ^44^ ^]^

The DNA carriers and protein analytes were incubated at 37 °C for 2 h before nanopore measurements. Under identical conditions, control experiments with pure VEGF and PDGF showed no detectable translocation events (Figure S12). However, dimeric events with doubled dwell times and charges were observed upon protein addition for both VEGF and PDGF (Figure 4a,b). Notably, these events featured centrally located subpeaks, further confirming the successful formation of aptamer‐protein‐aptamer sandwich complexes.

Charge versus dwell time scatter plots for VEGF and PDGF, shown in Figure 4c, highlight the dimeric events. Further subpeak analysis of the extracted dimeric events is depicted in Figure 4d. The mean fractional positions for VEGF and PDGF were found to be 0.59 and 0.57, respectively. The mean normalized subpeak amplitude for VEGF (0.879) was higher than for PDGF (0.817), which correlates with the size of the respective proteins. Binding assays for both VEGF and PDGF showed characteristic two‐phase transitions, with *K*_d_ values of 0.3 pM and 1.8 pM, respectively.

### Direct Observation of the Activation and Inhibition of c‐Met Dimerization

This strategy was further explored for its ability to monitor dynamic protein dimerization, specifically focusing on receptor protein dimerization induced by ligand binding. Receptor protein dimerization plays a fundamental role in initiating intracellular signal transduction, which is essential for normal biological processes and is implicated in disease development, including cancer.^[^ ^45^ ^]^ Receptor tyrosine kinases (RTKs) are a class of transmembrane proteins expressed widely. They serve as receptors for various extracellular signaling molecules, such as growth factors and neurotrophic factors, regulating essential cellular activities.^[^ ^46^ ^]^ The canonical model of RTK activation suggests that ligand binding shifts the equilibrium from inactive monomeric receptors to active receptor dimers.^[^ ^47^ ^]^ This dimerization, along with intracellular domain phosphorylation, is a key step in triggering a cascade of intracellular signaling events.^[^ ^48^ ^]^ Therefore, understanding the expression levels and oligomerization states of RTKs is crucial for comprehending the intricacies of signal transduction networks. To validate this concept, we chose the c‐Met receptor (130 kDa) as a model, which acts as the cognate receptor for hepatocyte growth factor (HGF, 80 kDa).^[^ ^49^ ^]^ HGF activates the c‐Met receptor by promoting receptor dimerization upon binding.^[^ ^50^ ^]^ For this proof‐of‐concept study, we employed a 40‐mer DNA aptamer with an affinity of approximately 0.09 nM to specifically target the c‐Met receptor.^[^ ^51^ ^]^

**Figure 5:**
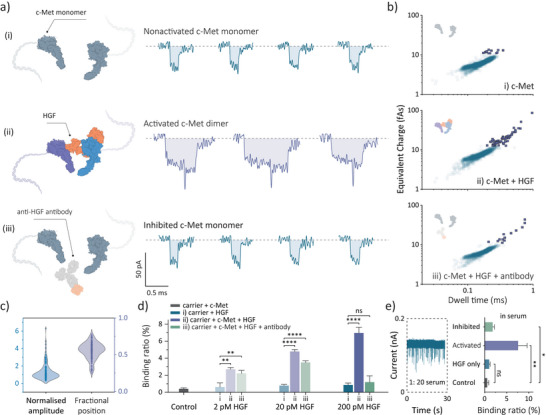
Nanopore detection of HGF‐induced c‐Met dimerization using aptamer‐modified DNA carriers. a) Schematic and typical events for (i) monomeric c‐Met receptors bound to the DNA carriers, (ii) HGF‐induced c‐Met dimers bound to two DNA carriers, and (iii) c‐Met receptors and HGF under the inhibition of anti‐HGF antibody. b) Scatter plots of charge against dwell time for (i) non‐activated c‐Met monomers (200 pM carrier + 200 pM c‐Met) (*n* = 2196), (ii) activated c‐Met dimers (200 pM carrier + 200 pM c‐Met + 200 pM HGF) (*n* = 1808) and (iii) inhibited c‐Met monomers (200 pM carrier + 200 pM c‐Met + 200 pM HGF + 5 nM anti‐HGF antibody) (*n* = 1279), where the dimeric events over the charge threshold were highlighted with purple color, respectively. Representative events were collected with 200 pM protein concentration, 5 nM anti‐HGF antibody concentration and 200 pM carrier concentration in 100 mM KCl measuring buffer under a bias of −300 mV. c) The distribution of normalized subpeak amplitude and fractional position for dimeric events extracted from the translocation of HGF‐induced c‐Met dimers bound to DNA carriers (*n* = 168). d) Dimerization ratio of c‐Met in the presence of varying HGF concentrations (0, 2, 20, and 200 pM). DNA carrier and c‐Met concentration were fixed to be 200 pM and an excess concentration of 5 nM anti‐HGF antibody were used to monitor the inhibitory effect. T scores were used to test the statistical significance between different groups; ns *P* > 0.05, *P ≤ 0.05, **P ≤ 0.01, ***P ≤ 0.001 and ****P ≤ 0.0001. The error bars represent one standard deviation of at least three independent experimental repeats. e) Typical trace of DNA translocation in 1: 20 serum solution. Binding ratio of c‐Met dimers to DNA carrier (HGF: 200 pM) in 1: 20 serum solution. T scores were used to test the statistical significance between different groups (control: 200 pM DNA carrier + 200 pM c‐Met, HGF only: 200 pM DNA carrier + 200 pM HGF, activated: 200 pM DNA carrier + 200 pM c‐Met + 200 pM HGF, inhibited: 200 pM DNA carrier + 200 pM c‐Met + 200 pM HGF + 5 nM anti‐HGF antibody); ns *P* > 0.05, *P ≤ 0.05, **P ≤ 0.01 and ***P ≤ 0.001. The error bars represent one standard deviation of at least three independent experimental repeats.

Before utilizing DNA carriers, we conducted experiments with pure proteins, including the c‐Met receptor alone, the c‐Met receptor with HGF, and the c‐Met receptor with HGF in the presence of an anti‐HGF antibody. Under low salt concentration conditions, no observable translocation events were detected for these pure protein samples, as shown in Figure S13. To assess specificity and sensitivity, we performed control experiments using aptamer‐modified DNA carriers with monomeric c‐Met receptors, confirming that c‐Met did not self‐dimerize under the given conditions. However, upon HGF addition, c‐Met receptors formed homodimers, as indicated by the translocation events of dimerized DNA, Figure 5a. This approach was also sensitive enough to detect changes in dimerization when the c‐Met receptors were treated with an anti‐HGF antibody, which inhibited HGF activity. Figure 5b presents scatter plots of charge versus dwell time for non‐activated, activated, and inhibited c‐Met receptors, providing a direct visualization of the varying proportions of dimerized signals.

DNA carriers transporting large protein complexes, such as HGF‐induced c‐Met dimers (340 kDa), exhibited translocation behavior similar to previous DNA‐protein complexes, with a characteristic subpeak at ≈0.58 fractional position. The normalized subpeak amplitude correlated directly with protein size, Figure S14, with c‐Met dimers showing significantly higher amplitudes than smaller proteins.

We also examined the effect of varying HGF concentrations on c‐Met dimer formation, Figure 5d. As HGF levels increased from 2 to 200 pM, the proportion of dimeric c‐Met events rose, while the inhibitory effect of excess anti‐HGF antibodies became more pronounced. This trend aligns with fluorescence‐based observations from previous studies.^[^ ^52^ ^]^ For clinical applications, it is essential that the nanopore biosensing approach is compatible with unprocessed biological samples.^[^ ^53^ ^]^ Human serum, a common clinical biofluid, contains a diverse range of proteins that could introduce background interference. Under low salt conditions, however, most serum proteins produced no detectable translocation signals despite their size. To maintain a stable current baseline, we optimized a serum dilution ratio of 1:20 (serum: buffer, v/v), achieving a baseline noise level comparable to that of the measuring buffer alone, Figure S15. The optimal dilution ratio was determined to be 1: 20 (serum: buffer, v/v), which yielded a noise level comparable to that of the measuring buffer alone. Subsequent c‐Met activation and inhibition experiments in diluted serum yielded results consistent with those in the measuring buffer, demonstrating the platform's potential for selective protein detection in complex clinical samples, Figure 5e.

## Conclusion

This study introduced a versatile nanopore‐based platform for protein identification and quantification using aptamer‐modified DNA carriers. Instead of directly analyzing subpeak information, the approach detects proteins indirectly through DNA carrier dimerization. The unique charge signatures of dimeric DNA carriers allow straightforward classification, making the method applicable to any protein with two distinct aptamer‐binding sites. We demonstrated ultrasensitive thrombin detection using HD1‐ and HD22‐modified DNA carriers, confirming aptamer‐protein sandwich formation through characteristic dwell times, charge distribution, and centrally located subpeaks. This method was extended to detect small dimeric proteins, such as VEGF and PDGF, using identical DNA carriers. Additionally, we successfully monitored ligand‐induced receptor dimerization, specifically HGF‐induced c‐Met dimerization, demonstrating the platform's adaptability for studying dynamic protein interactions. Even at near‐physiological salt concentrations, the aptamer‐protein complex produced a distinct subpeak, offering insights into protein size and shape. We confirmed compatibility with complex biological fluids, including human serum, highlighting the potential for diagnostics using clinical samples.

To further enhance its diagnostic utility, particularly in oncology and precision medicine, it is important to address challenges such as differentiating protein isoforms with subtle subunit variations and detecting rare variants in complex biological environments. While our approach provides high specificity, it could be complemented with recent advances in nanopore technology that enable subunit‐level discrimination, allowing finer resolution of protein heterogeneity.^[^ ^54^, ^55^ ^]^ Furthermore, improvements in signal deconvolution and real‐time classification could expand this platform to profile complex clinical samples such as serum or cerebrospinal fluid (CSF), where rare or low‐abundance biomarkers may be masked. Combining these advances could pave the way for next‐generation single‐molecule biomarker profiling directly from unprocessed clinical samples.

## Conflict of Interests

The authors declare no conflict of interest.

## Supporting information



Supporting Information

## Data Availability

The data that support the findings of this study are available from the corresponding author upon reasonable request.
