# Supplementary material for: Single‐Molecule Protein Profiling Using Nanopores and Dimeric Aptamer‐Modified DNA Carriers
Source: Angew Chem Int Ed Engl. 2025 Aug 15;64(39):e202505902. doi: 10.1002/anie.202505902 (PMC12455474; doi:10.1002/anie.202505902)
Supplement: Supplementary file 1 — Supporting Information [file ANIE-64-e202505902-s001.pdf]

Supporting Information

Single-Molecule Protein Profiling Using Nanopores and Dimeric Aptamer-Modified DNA Carriers

Xiaoyi Wang,[a][c]# Yaxian Liu,[a]# Ren Ren,[a][b] Joshua B. Edel,[a] and Aleksandar P. Ivanov*[a]

# These authors contributed equally to this work.

[a] Dr. X. Wang, Dr. Y. Liu, Dr. R. Ren, Prof. J. B. Edel, Prof. A. P. Ivanov
Department of Chemistry, Imperial College London, Molecular Sciences Research Hub, White City Campus, 82 Wood Lane, London W12 0BZ, UK
E-mail: joshua.edel@imperial.ac.uk; alex.ivanov@imperial.ac.uk

[b] Dr. R. Ren
Department of Metabolism, Digestion and Reproduction, Imperial College London, Hammersmith Campus, Du Cane Road, London W12 0NN, UK

[c] Dr. X. Wang
Picower Institute for Learning and Memory, Massachusetts Institute of Technology (MIT), Cambridge, MA 02139, USA

EXPERIMENTAL

Preparation of aptamer-modified DNA carriers

The 4.3 kbp aptamer-modified DNA carriers were prepared by digesting lambda phage DNA (λ-DNA, 48.5 kbp) with the restriction enzyme HindIII, followed by integration of the aptamer probe into the sticky overhangs of the 4.3 kbp fragments. The fragments were then separated and purified using agarose gel electrophoresis. 12.5 µl of commercial λ-DNA (15.8 nM, New England Biolabs, UK) was mixed with 2.5 µl HindIII-HF (New England Biolabs, UK), 5 µl of 10× rCutSmart buffer (New England Biolabs, UK), and 30 µl of nuclease-free water (Thermo Scientific, US). The mixture was incubated at 37 °C for 60 min, followed by thermal inactivation at 80 °C for 20 min. After digestion, 1 µl of a 100 µM phosphorylated DNA aptamer solution (Integrated DNA Technologies, IDT, US) was added to the mixture. A hybridization procedure was conducted using a PCR annealing device (TC-3000, TECHNE) with the following customized protocol: the mixture was initially heated to 75 °C for 5 min, then gradually cooled to 15 °C at a rate of 1 °C/min, and finally held at 4 °C. The aptamer sequences are listed in Table 4.1. The resulting product was ligated by adding 2.5 µl of T4 DNA ligase (400 units/µl, New England Biolabs, UK) and 6 µl of 10× T4 DNA ligase reaction buffer (New England Biolabs, UK). Ligation was performed at 22 °C for 2 hours, followed by inactivation at 65 °C for 15 min. To purify the final product, the ligated DNA carriers were separated by preparative gel electrophoresis and then extracted using the Monarch DNA Gel Extraction Kit (New England Biolabs, UK). The final concentration of the carrier was determined by measuring absorption at 260 nm using the NanoDrop 2000 (Thermo Scientific, US).

Fabrication of nanopipettes

For the fabrication of single-barrel nanopipettes, quartz capillaries (GQF100-50-7.5, World Precision Instruments, UK) were initially subjected to plasma cleaning (Harrick Plasma, US) for at least 30 minutes. The cleaned capillaries were then pulled using a laser-based pipette puller (Sutter Instrument, P-2000, USA) following a two-line protocol:Line 1: HEAT = 825, FIL = 4, VEL = 30, DEL = 130, PUL = 80;Line 2: HEAT = 850, FIL = 3, VEL = 20, DEL = 127, PUL = 185. These pulling parameters are instrument-specific and were carefully optimized to produce nanopore openings with a diameter of 15 ± 4 nm

Nanopore measurements and data processing

Nanopore experiments were performed in a measuring buffer containing 100 mM KCl and 10 mM Tris-EDTA (pH = 8) unless noted otherwise. For the binding assays, 200 pM aptamer-modified DNA carriers were incubated with the analyte protein with a gradient of concentrations at 37 °C for at least 2 h prior to nanopore measurements. Analyte proteins tested in this work include Recombinant Human VEGF 165 Protein (R&D Systems, US), Recombinant Human PDGF-BB Protein (R&D Systems, US), Human alpha-Thrombin (Prolytix, US), Recombinant Human HGFR/c-MET Fc Chimera His-tag Protein (R&D Systems, US),

Recombinant Human HGF Protein (R&D Systems, US) and Human HGF Antibody (R&D Systems, US). FDA-approved pooled human serum from human male AB plasma, USA origin, was purchased from Sigma Aldrich, H4522. Consent and ethical approvals were obtained by the provider. No further approvals were required for this study.

To perform nanopore measurements, approximately 10 µL of the buffer solution containing the DNA carriers and proteins was filled inside the nanopipette (*trans* chamber) via a MicroFil needle (MF34G, World Precision Instruments, UK). Freshly made Ag/AgCl electrodes were then inserted into the nanopipette (*trans* chamber) and the bath (*cis* chamber), respectively. All ionic current recordings were performed using a high-bandwidth amplifier VC100 (Chimera Instruments, US) with a sampling time of 0.24 µs. The recorded data were resampled to 1 MHz and low-pass filtered at 30 kHz. Analysis of all translocation events was performed using a MATLAB-based code, The Nanopore App, written by Professor Joshua B. Edel. A workflow of the analysis procedure is shown in Figure S16.

Following feature extraction, peak area was used for charge classification of DNA carriers. The distribution of equivalent charge was fitted using Gaussian function and a threshold was set at the mean (μ) + three standard deviations (3σ) of the monomeric DNA charge distribution. Events with a charge greater than this threshold were classified as dimeric events and considered indicative of protein binding. The binding ratio was defined as the number of protein-bound DNA carriers divided by the total number of carriers detected.

The binding curves were fitted using the biphasic Hill equation:

$$y = \frac{P_{\mathrm{m}}}{\left[1 + \left(\frac{K_{\mathrm{a}}}{x}\right)^{H_{\mathrm{a}}}\right]\left[1 + \left(\frac{x}{K_{\mathrm{i}}}\right)^{H_{\mathrm{i}}}\right]}$$

where P_{m} = maximum binding ratio, K_{a} = half-maximal activation concentration, K_{i} = half-maximal inhibition concentration, H_{a} = activation Hill coefficient, H_{i} = inhibitory Hill coefficient. K_{a} was used as K_{d} in our binding assay.

Table S1 Sequences of the aptamer probes used in this work.

Name	Sequences
Thrombin probe 1	5'-GGGCGGCGACCT TTTTTT GGTTGGTGTGGTTGG
Thrombin probe 2	5'-GGGCGGCGACCT TTTTTT AGTCCGTGGTAGGGCAGGTTGGGGTGACT
VEGF probe	5'-GGGCGGCGACCT TTTTTT TGTGGGGGTGGACGGGCCGGGTAGA
PDGF probe	5'-GGGCGGCGACCT TTTTTT CACAGGCTACGGCACGTAGAGCATCACCATGATCCTGTG
c-Met probe	5'-GGGCGGCGACCT TTTTTT TGGATGGTAGCTCGGTCGGGGTGGGTGGGTTGGCAAGTCT

The blue colored sequence is the complementary bases to the 12-base sticky end of the carrier DNA, and the red colored sequence is the aptamer probe targeting different proteins.

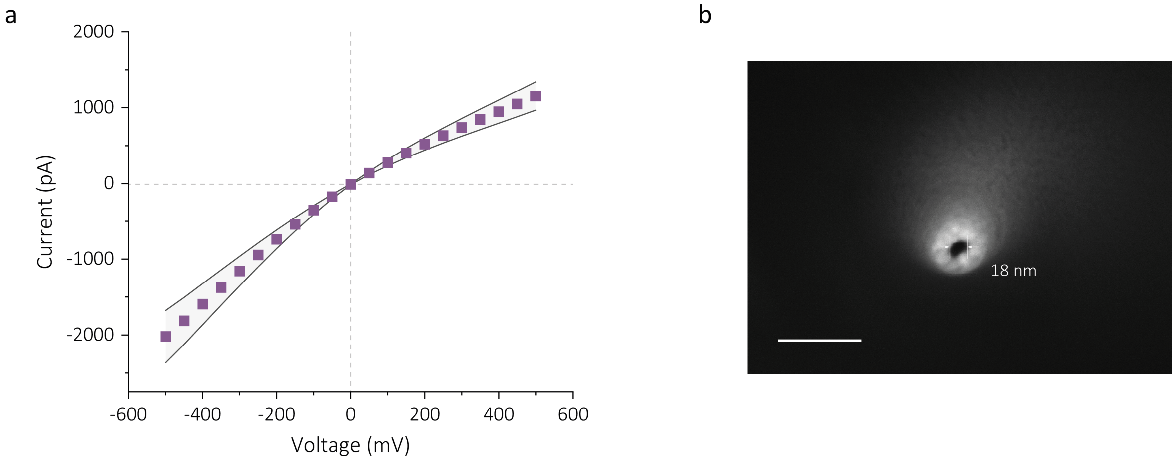

Figure S1 SEM and conductance characterization of nanopipettes. The nanopipettes used in this work were fabricated from quartz capillaries (ID: 0.5 mm, OD: 1.0 mm) by a laser-assisted puller. (a) I-V curves were measured from 20 different pipettes in 100 mM KCl, 10 mM Tris-EDTA, pH 8.0 buffer, showing a current rectification feature. (b) Top-view SEM images of a typical nanopore (scale bar: 100 nm).

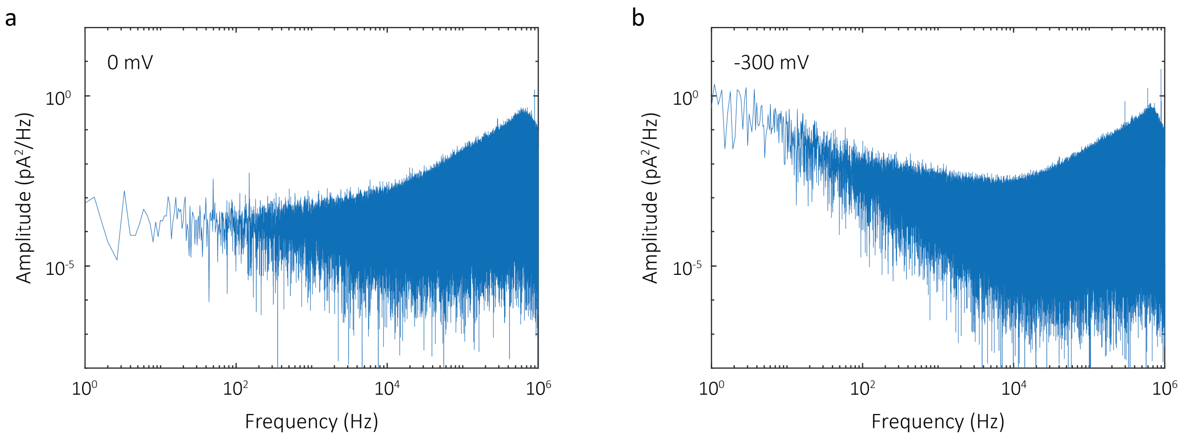

Figure S2 Noise evaluation for nanopore measurements. The power spectral density (PSD) plots for the nanopipette system under a bias of (a) 0 mV and (b) -300 mV.

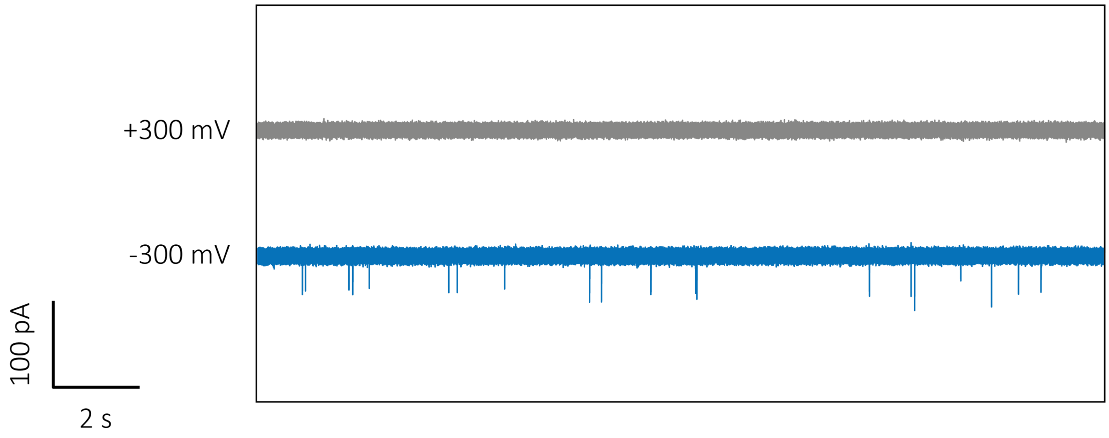

Figure S3 DNA translocation direction in the nanopore sensing. DNA molecules translocated from the inside to the outside of the nanopipette under a negative bias showing conductive enhancement events, whereas no translocation events were observed at positive voltages. The traces were collected from 200 pM 4.3 kbp DNA in 100 mM KCl, 10 mM Tris-EDTA pH 8.0 buffer with a voltage of ±300 mV.

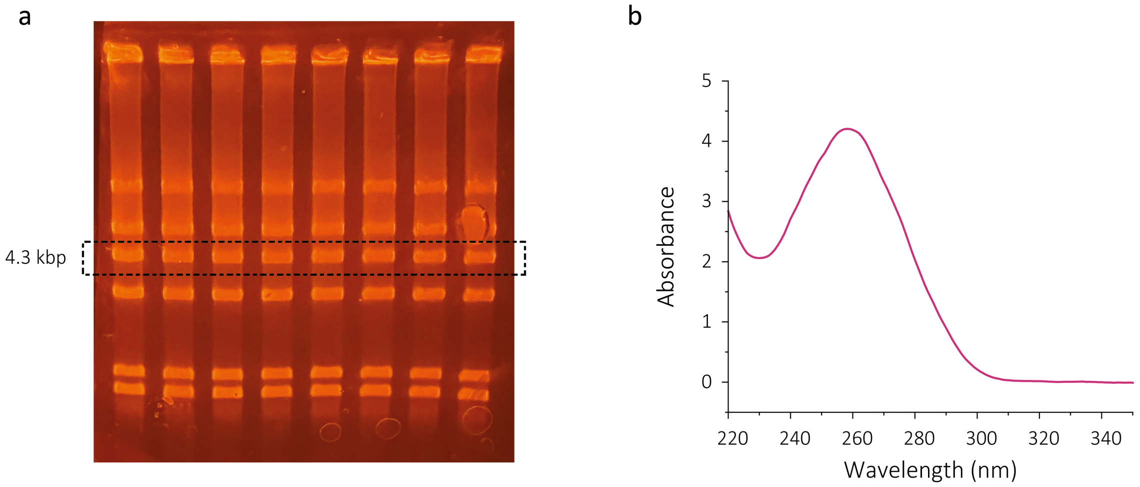

Figure S4 Characterization of 4.3 kbp aptamer-modified DNA carriers. (a) Gel electrophoresis showing the fragments digested from λ-DNA using Hind III. The marked bands are the expected 4.3 kbp DNA fragments. (b) A typical absorption spectrum of the purified 4.3 kbp aptamer-modified DNA carriers showing its concentration and purity with a high 260/280 ratio.

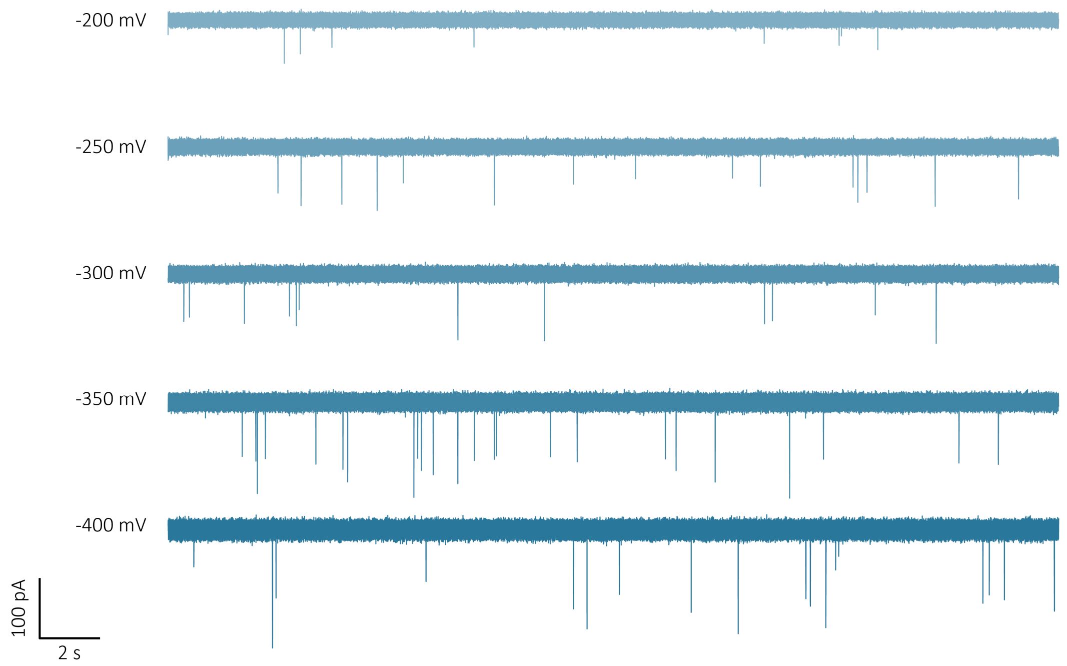

Figure S5 DNA translocation at varying applied voltages. Representative current traces at a gradient voltage showing the translocation of 4.3 kbp aptamer-modified DNA carrier in 100 KCl, 10 mM Tris-EDTA pH 8.0 measuring buffer.

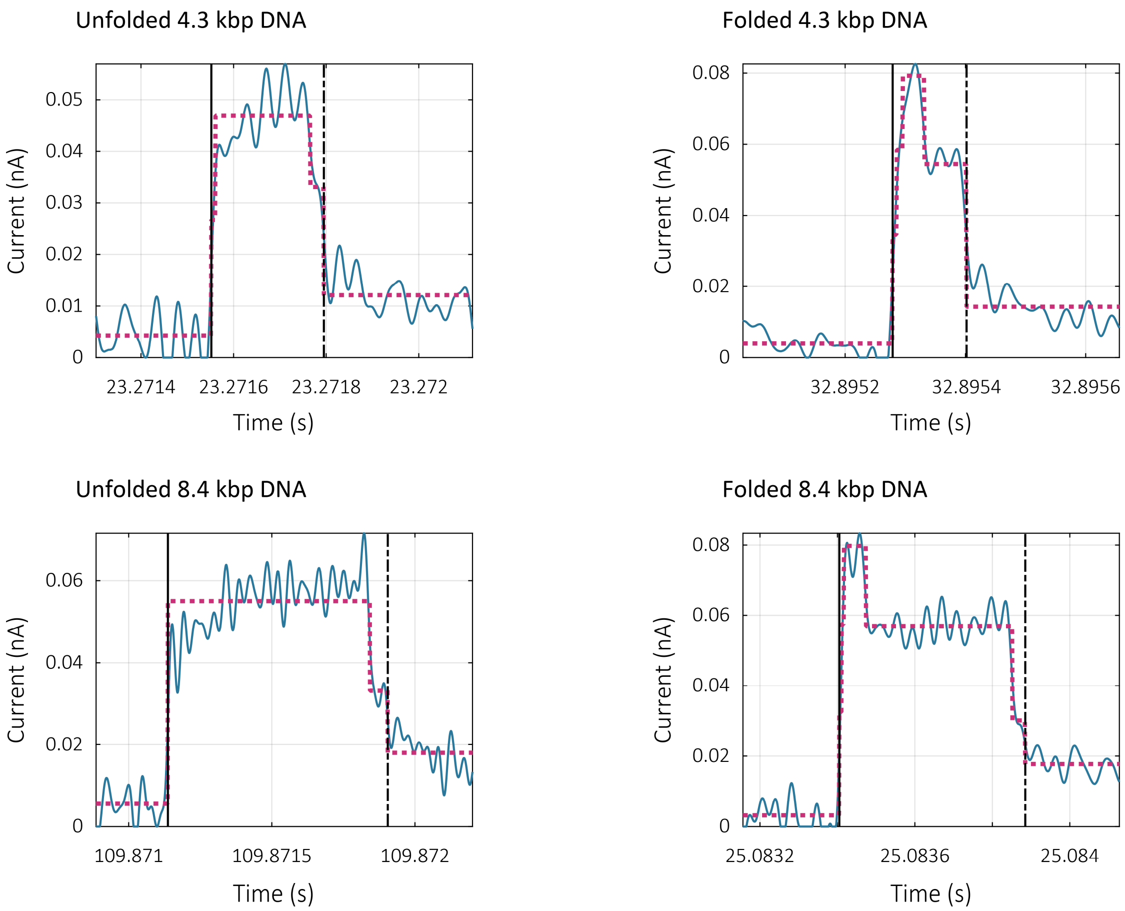

Figure S6 Event definition using CUSUM+ algorithm. Typical events for 4.3 kbp and 8.4 kbp DNA in both folded and unfolded states are shown in blue lines. The boundaries for event definition are established through the fitting of the CUSUM+ analysis. The equivalent charge is subsequently calculated based on the peak area within the boundaries determined by the CUSUM+ algorithm.

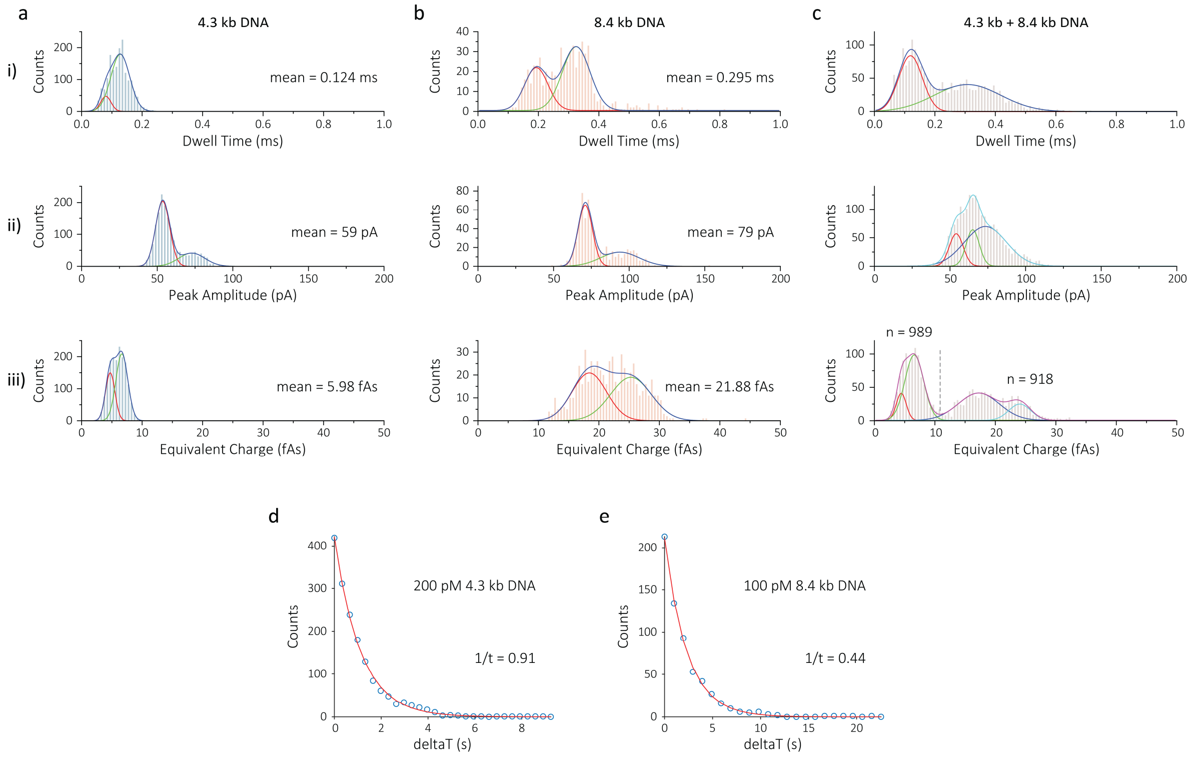

Figure S7 Statistical analysis of 4.3 kbp and 8.4 kbp DNA translocation. Histograms of (i) dwell time, (ii) peak amplitude and (iii) equivalent charge for (a) 200 pM 4.3 kbp DNA (*n* = 1625), (b) 100 pM 8.4 kbp DNA (*n* = 621) and (c) mixture of 100 pM 4.3 kbp DNA and 100 pM 8.4 kbp DNA (*n* = 1907), along with multiple peak Gaussian fittings. Nanopore experiments were performed in the measuring buffer containing 100 mM KCl and 10 mM Tris-EDTA pH 8.0 under a bias of -300 mV. Distributions of the inter-event time (the time between successive captured events) for (d) 200 pM 4.3 kbp DNA and (e) 100 pM 8.4 kbp DNA at an applied voltage of -300 mV. Solid lines represent single-exponential fits used to extract capture rate constants. The normalized capture rate of 4.3 kbp (4.5 events$\cdot s^{-1}\cdot nM^{-1}$) and 8.4 kbp (4.4 events$\cdot s^{-1}\cdot nM^{-1}$) DNA are very similar under these measuring conditions. This similarity is further observed by the event counts in the DNA mixture (panel c(iii)), where similar numbers of events were detected for each DNA species.

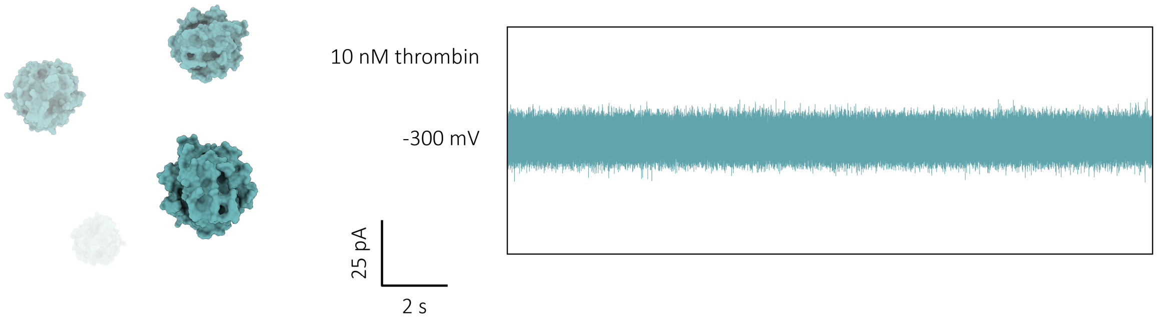

Figure S8 Nanopore measurements of pure thrombin. Nanopore experiments were performed using 10 nM thrombin in 100 mM KCl measuring buffer under a bias of -300 mV, without translocation events detectable under this condition.

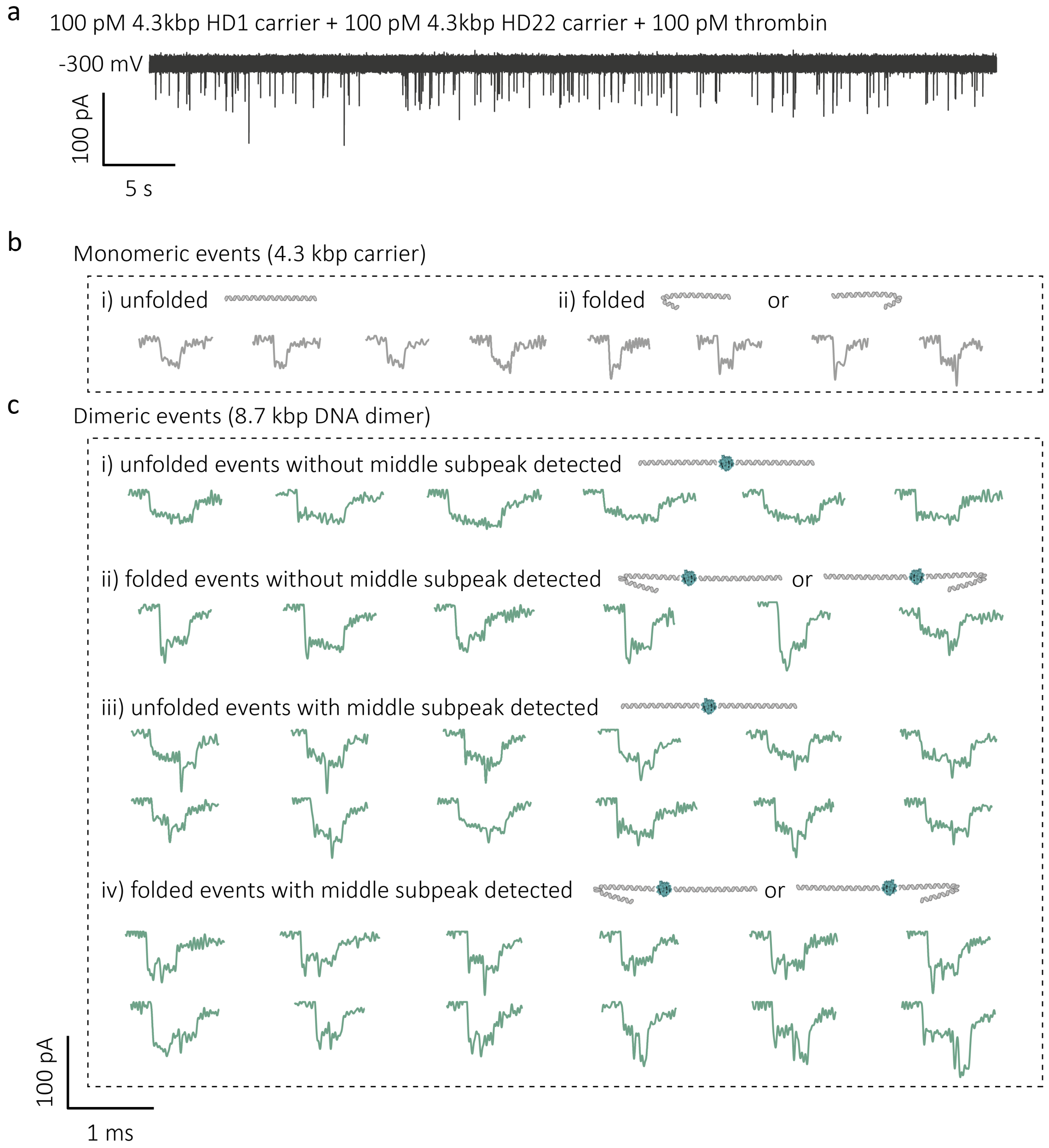

Figure S9 Representative current traces and events of thrombin detection. (a) A typical ionic current trace recorded in the presence of 100 pM thrombin and 100 pM DNA carrier in the 100 mM KCl measuring buffer under a bias of -300 mV. (b) Monomeric events: (i) unfolded events without any detectable subpeaks; (ii) folded events with subpeaks at either the beginning or end indicating the DNA folding states. (c) Dimeric events with sandwich structure formed: (i) unfolded events with no subpeak detectable in the middle; (ii) folded events with subpeaks at either ends (DNA folding), but no subpeak in the middle; (iii) unfolded events with subpeaks in the middle (detectable protein binding), but no subpeaks at the ends; (iv) folded events with subpeaks at the ends (DNA folding) and in the middle (protein binding).

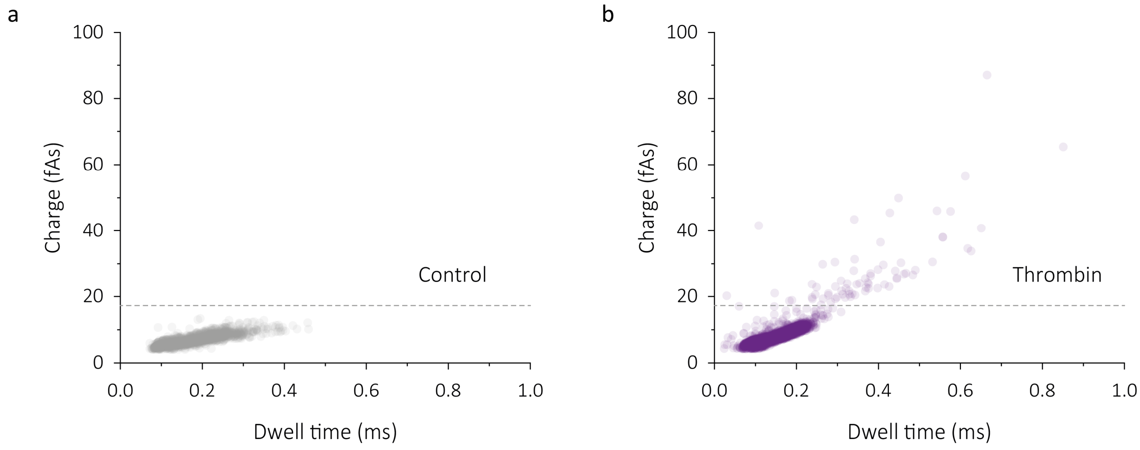

Figure S10 Statistics of carrier translocation with and without thrombin. Scatter plots of charge versus dwell time for (a) 100 pM HD1-modified carrier and 100 pM HD22-modified carrier; (b) 100 pM thrombin incubated with 100 pM HD1-modified carrier and 100 pM HD22-modified carrier. Nanopore experiments were performed in 100 mM KCl measuring buffer under a bias of -300 mV.

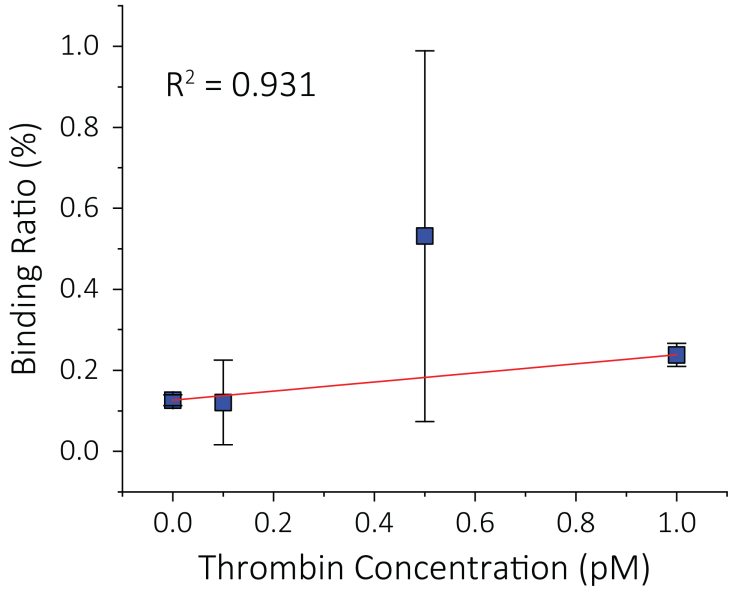

Figure S11 Calculation of limit of detection (LOD) for thrombin binding assay. Four data points at the low concentration region were fitted using linear regression. LOD = $3\sigma/m$ was used to calculate the limit of detection where σ is the standard deviation of blank measurements and m is the slope of the fitted curve. LOD was determined to be 0.20 pM in this case.

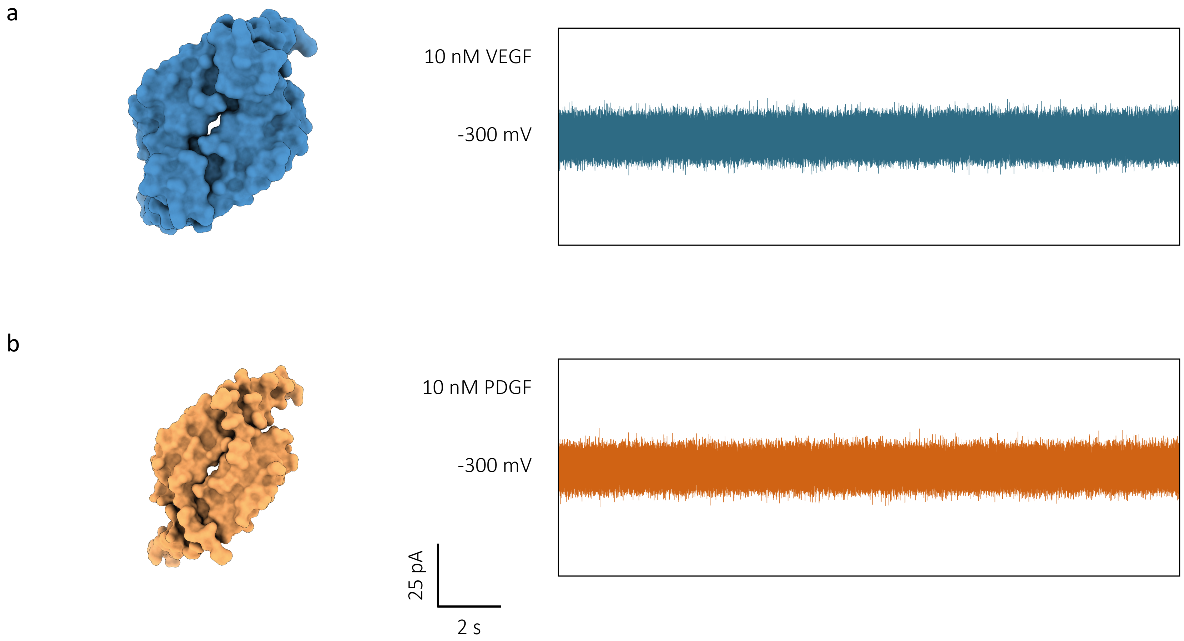

Figure S12 Nanopore measurements of pure VEGF and PDGF. Nanopore experiments were performed using 10 nM VEGF/PDGF in 100 mM KCl measuring buffer under a bias of -300 mV, without translocation events detectable under this condition.

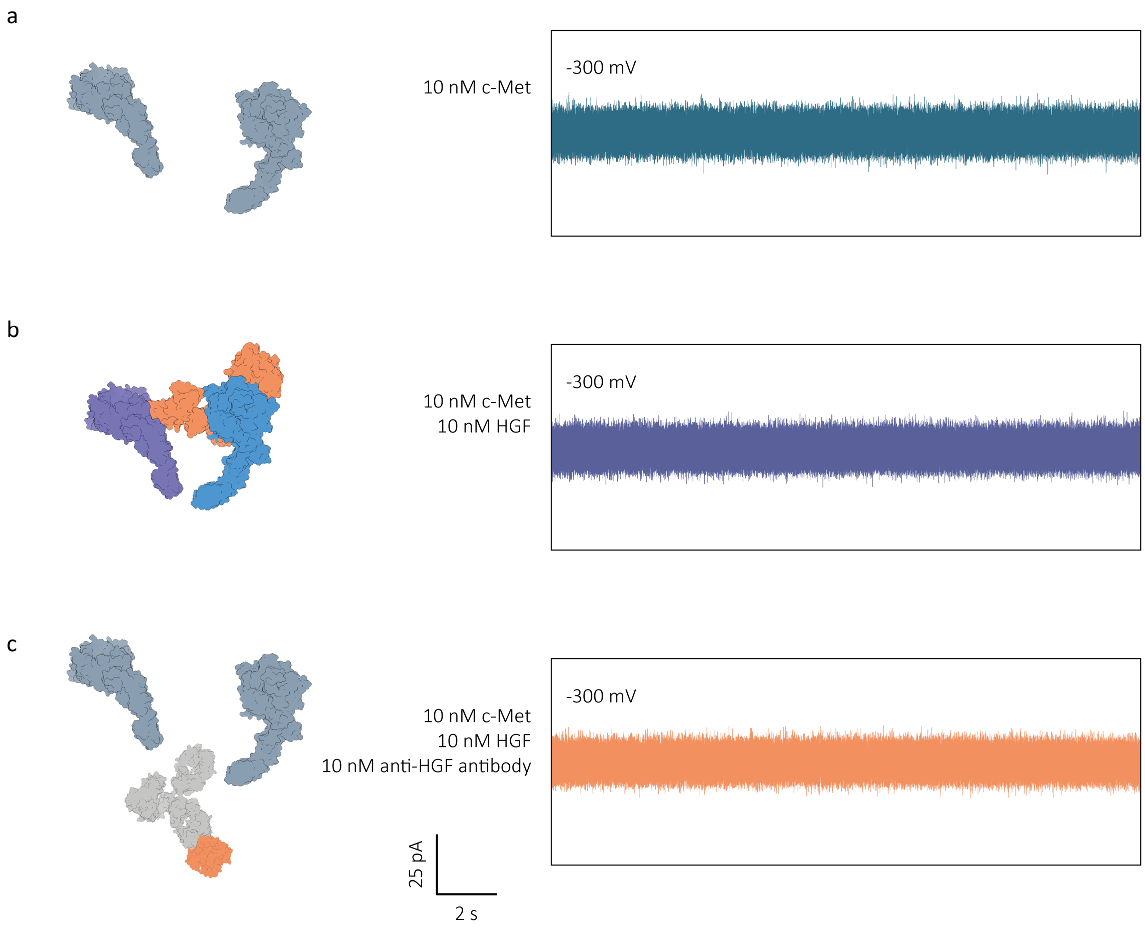

Figure S13 Nanopore measurements of pure c-Met. Nanopore experiments were performed using (a) 10 nM c-Met, (b) 10 nM c-Met + 10 nM HGF, and (c) 10 nM c-Met + 10 nM HGF + 10 nM anti-HGF antibody in 100 mM KCl measuring buffer under a bias of -300 mV, without translocation events detectable under this condition.

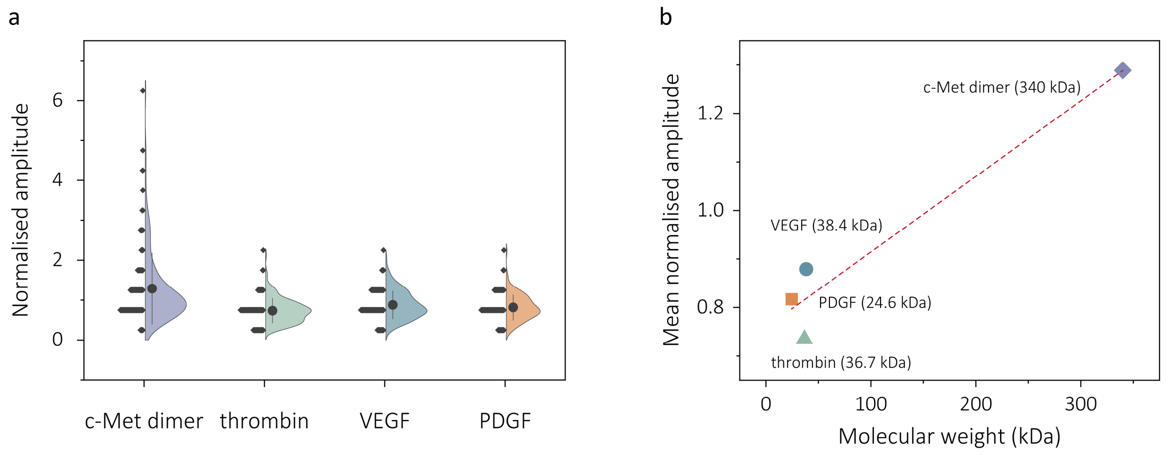

Figure S14 Subpeak analysis for translocation events of HGF-induced c-Met dimerization. (a) The distribution of normalized subpeak amplitude and fractional position for dimeric events extracted from the translocation of HGF-induced c-Met dimers bound to DNA carriers. (b) Comparison of the distribution of normalized subpeak amplitude for the c-Met dimer, thrombin, VEGF and PDGF. (c) A linear correlation was shown on mean normalized subpeak amplitude against the molecular weight.

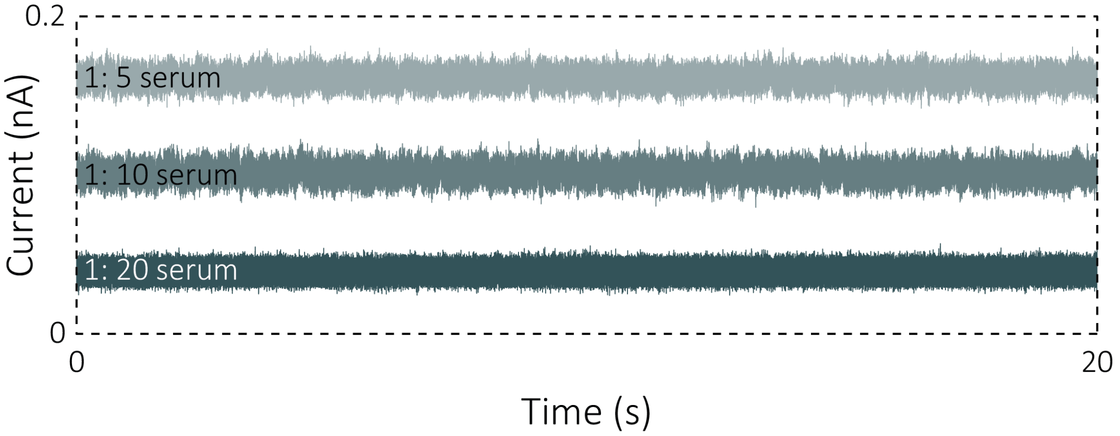

Figure S15 Nanopore measurements in human serum. Current baseline for the dilution of human serum to the measuring buffer (1: 5, 1: 10 and 1: 20).

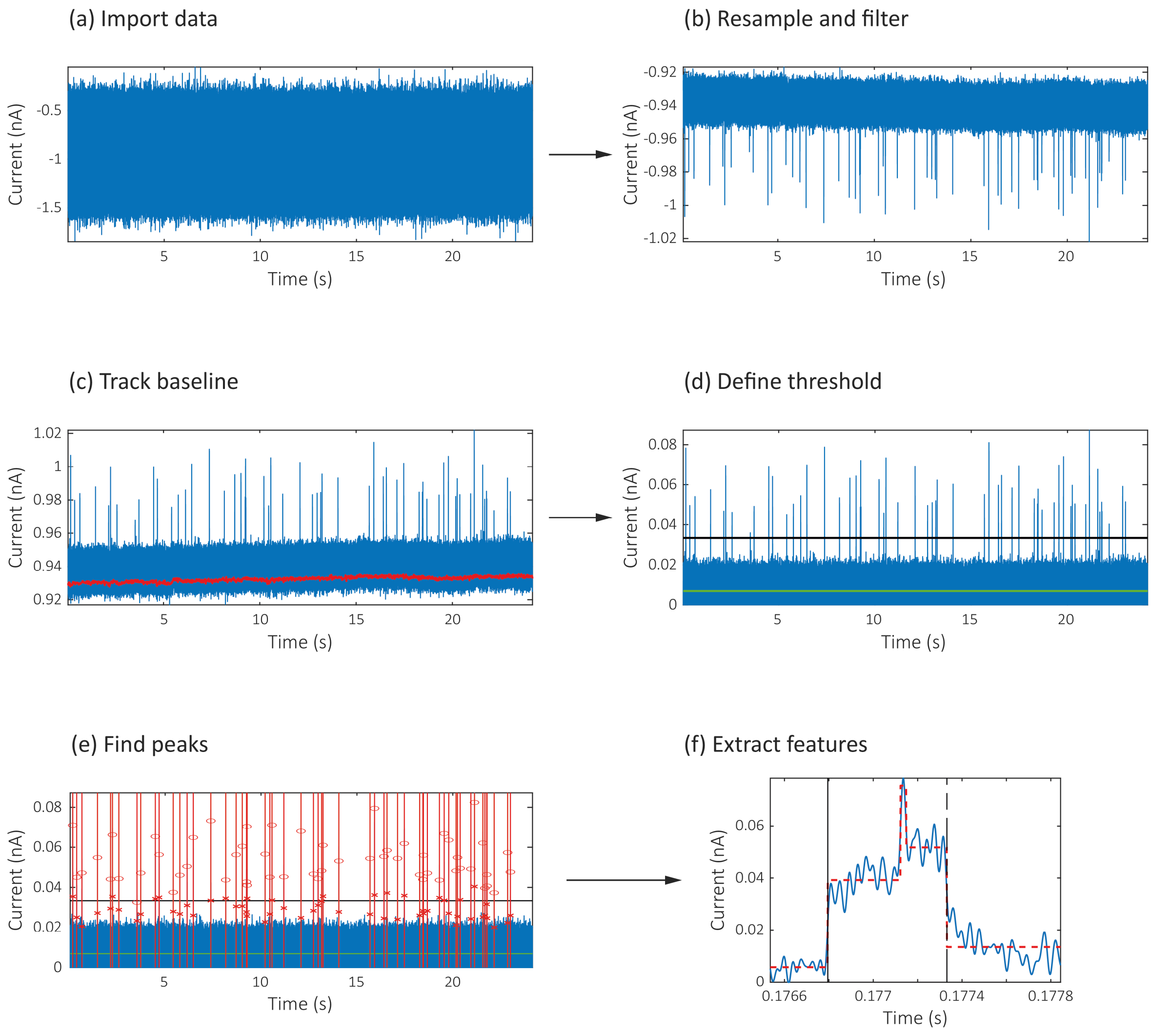

Figure S16 Workflow for data analysis. (a) Data import. In this step, nanopore data was loaded into the Nanopore App with a sampling rate of 0.24 µs. (b) Data resampling and filter. Next, the current-time data was resampled and low-pass filtered dependent on the signal-to-noise ratio. In this work, a resampling rate of 1 MHz and a low-pass filter of 30 kHz were used. (c) Baseline tracking. The baseline was tracked to compensate for fluctuations in the current recordings. (d) Thresholding. A histogram of all points was fitted using a Poisson distribution for the baseline, and a threshold (e.g., 8 σ) was used to define the translocation events. (e) Peak selection. Events with a peak amplitude above the defined threshold were selected. (d) Feature extraction. Individual events were fitted using the CUSUM+ algorithm. As a result, event features such as amplitude, dwell time, charge and subpeak information could be exported.
